# Elemental Contents in Hair of Children from Two Regions in Dar Es Salaam

**DOI:** 10.1155/2012/495043

**Published:** 2012-03-18

**Authors:** Najat K. Mohammed

**Affiliations:** Department of Physics, University of Dar es Salaam, P.O. Box 35063, Dar es Salaam, Tanzania

## Abstract

The work presented in this paper is part of the study which aims at determining the levels of elements in hair of children in Tanzania as a bioindicator of their nutrition and health. In this paper, the levels of trace elements in hair from children living in Dar es Salaam have been analysed. The analysis was carried out by long and short irradiation INAA at the reactor centre of the Institute of Nuclear Physics, Rez Czech Republic. 22 samples were collected from children living at Kiwalani about 12 km from Dar es Salaam city and 16 samples from children living at Mlimani, the main campus of University of Dar es Salaam. A total of 34 elements were found in the hair of the children. There were no big differences between the concentration levels of the essential elements in hair samples collected from the children which might indicate the same food consumption habits.

## 1. Introduction

Trace element concentrations in human body are normally determined by the analysis of blood, serum, plasma, urine, or hair. Hair has an advantage over blood and urine as it accumulates elements in higher concentrations [1] and its collection is fast, painless, and easy [2]. Further more, the analysis of hair gives long-term information on the behaviour of the elements in the body (up to 2-3 years depending on length of hair) in contrast to blood and urine which provide short-term information [3]. Hair is also a stable biological material, which can easily be stored at room temperature for a long time without a change in its composition.

A number of researchers have used hair samples to assess the nutritional and environmental exposure to humans. Othman and Spyrou (1980) reported increased levels of mercury (Hg) in hair samples collected from female adults and children in the Machakos district in Kenya, which are associated with the use of Hg compounds as cosmetics and for lightening the colour of the skin [4]. Airey [5], analysed human hair samples for Hg in relation to fish consumption. The results showed a direct relationship between mean hair Hg and the rate of fish consumption and hence to the diet of the society. Ikingura et al. (1996) reported concentrations of Hg in hair samples which were consistent to the concentration levels found in fish consumed by people living in the Lake Victoria goldfields in Tanzania [6]. Fordyce et al. (2000), reported a clear link between dietary Se intake and its concentrations in hair [7]. Hair from children has also been used in several research projects to assess their health and nutritional status [8–12]. The effect of dietary factor, geographical locations, and environmental exposure on the hair elemental concentration of children from Tanzania has been presented in a previous paper [13].

In this study, hair samples of children from two socioeconomic groups in Dar es Salaam have been analysed.

## 2. Sample Collection

 A total of 34 samples of hair from children of age between 5–15 years (Average 10 years) were collected from two regions of Dar es Salaam representing two socioeconomic classes. 22 samples were collected from children living at Kiwalani about 12 km from Dar es Salaam city and 16 samples from children living at Mlimani, the main campus of University of Dar es Salaam. The children from Kiwalani had parents with low income and primary school education whilst children from Mlimani were from the academic staff of the University of Dar es Salaam. In this paper, the former group will be considered as the low class while the later group as the middle-class children.

About 70 to 500 mg of hair was cut using precleaned stainless steel scissors as near as possible to the scalp from the left side of the nape. All children or the parents (when a child was below 7 years) were interviewed at the time of sampling to obtain some general information on their health. The hair samples were then cut into 1 cm long strands put into plastic bags and kept in desiccators.

## 3. Sample Preparations

The hair samples were washed according to methods proposed by the IAEA 1977 [14]. The samples were then air dried for a minimum of 48 hours in a clean room laboratory and then inserted into a Polyethylene disc and sent to the Rez reactor centre for irradiation.

## 4. Sample Analysis

The irradiations were carriedout in the nuclear reactor LVR-15 of the Nuclear Research Institute Řež, operated at 9 MW. For short irradiation, samples and standards were irradiated in the active core of the nuclear reactor. The samples and standards were irradiated separately in polyethylene (PE) rabbits for 1 min, waited for 10 min before counted for 10 min. After irradiation, the capsules containing the samples were surface washed with water and ethyl alcohol and counted using a well-shielded n-type hyper pure germanium detector (HPGe) of relative efficiency 20.8%.

For intermediate and long-lived irradiations, the samples and standards were irradiated in a water-cooled Al can in a channel located at the outskirts of the reactor core with neutron fluence rate 8 × 10^13^ and 2 × 10^13^ n cm^−2^ s^−1^ for thermal and fast neutrons, respectively. The samples were irradiated for 2 hours, followed by 4 days of initial decay and 30 min counting time. Further 2 hours counting was carried out after 1 month decay. A total of 40 samples and standards each wrapped in Al foil were placed in one container of 9.5 cm length to form a column and the neutron flux monitors (Fe disc of about 60 mg and 10 mm in diameter) were inserted between each set of 5 samples with the standards. After the irradiation, samples and standards were counted in a shielded p-type HPGe detector of relative efficiency 20.8%.

 The concentration levels of the elements were calculated by the comparative method of INAA, using multielement standards (MES) [15]. Quality control was carried out using certified hair reference material GBW 07601 (National Research Centre for Certified Reference Materials, China), NIST Orchid Leaves (SRM 1571), Apple Leaves (SRM 1515), and Urban Particulate (SRM 1648) analysed with the samples. As Table 1 shows, the experimental values were all in good agreement with the certified values within 16% deviation.

## 5. Results and Discussion

Twenty nine elements which are Ag, Al, As, Au, Ba, Br, Ca, Cd, Ce, Cl, Co, Cr, Cu, Eu, Fe, I, K, Mg, Mn, Na, S, Sb, Sc, Se, Sm, Sr, U, V, Zn were detected in hair samples of children living in two regions of Dar es Salaam (Table 2). The data from the children living at Mlimani, the University of Dar es Salaam (middle class) were compared with the data from the children living in Kiwalani (low class). The Mann-Whitney test indicated that children from Mlimani had significantly (*P* < 0.05) higher concentrations of Al, K, Sr, and Sc whilst children from Kiwalani had higher concentrations of I. The essential elements Zn in hair from both groups were similar (~105 *μ*g/g) whilst Fe and Cu were slightly but not significantly (19% and 27%, resp.) higher in children from Mlimani than children of the other group.

The mean concentrations for Zn and Cu in both groups were lower than the mean concentrations of these elements in children of the same age group reported in Kazakhstan, Hong Kong, and Italy [10–12]. Hambidge et al. (1976), reported Zn concentrations in the range of <105 *μ*g/g in the hair of low-income children who had their hair Zn concentration increased by 71% after 3 month of being provided with rich in Zn meals [8]. In this study, 41% and 58% of the samples from Kiwalani and Mlimani, respectively, had concentration below 105 *μ*g/g. The mean concentration of Cu from both groups were lower than the concentrations reported in Kazakhstan, Hong Kong, and Italy, although these values were within the range for normal concentration of hair Cu in healthy children (6−23 *μ*g/g) reported by Weber et al. [9]. Nevertheless, 14% and 8% of the samples from Kiwalani and Mlimani, respectively, had concentrations of less than 6 *μ*g/g, which Weber et al. (1990) obtained in the malnourished children [9].

There were no statistical differences in the means of the essential elements Fe, Zn, and Cu between the two groups which might indicate similar consumption habits. Most of the Tanzanian diets include cereals (rice and maize) as the main dish and beans and green leaves are taken as sauces. Normally the bioavailability of essential elements such as Fe and Zn is low in cereals and food of plant nature because these foods have high contents of phytic acid as well as fibers and polyphenols, which form insoluble salts with Fe and Zn and thus hindering their absorption [16]. The absorption of Fe and Zn could be enhanced if a meal is taken with a portion of animal protein because these proteins prevent the elements from forming the insoluble phytates with the phytic acid in the foods [17].

Since there were no big differences on essential elemental concentration between the two groups, the observation demonstrated that residents of Dar es Salaam irrespective of their classes have the same food consumption habits. Although the number of samples (22 for low class and 16 for middle class) was not big enough to come to a definite conclusion, there is a need for nutrition awareness and diet planning for both groups especially as the middle class group could afford to have additional animal protein in their meals to enhance the absorption of Fe and Zn. Education programmes of food and nutrition are an alternative way of promoting nutritional awareness in a population. This method could be effective in any society because malnutrition is not always a result of not having enough food; sometimes it is just on how to turn the foods into nutritious meals.

## 6. Conclusion

The results in this study showed no big differences between the means of the essential elements Fe, Zn, and Cu in hair samples collected from children in the two socioeconomic classes living in Dar es Salaam. This observation might indicate the same food consumption habits. The mean concentrations for Zn and Cu in both groups were lower than the mean concentrations of these elements in children of the same age group reported in Kazakhstan, Hong Kong, and Italy [10–12]. Although the number of samples was not big enough to come to a definite conclusion, the socioeconomic classes of children in Dar es Salaam did not have much influence on their hair elemental concentrations. Since the concentration of hair Zn has been associated with nutrition, poverty, and/or cultural habits of the people [18], education on nutrition awareness and diet planning is needed for all socio groups in Tanzania especially as the high-income group could afford to have balanced diet.

## Figures and Tables

**Table 1:** The concentration levels of elements (*μ*g/g) in four-standard reference materials obtained in this work (Exp.) together with the certified values (Cert.).

	Hair (GBW07601)		Urban particulate (SRM 1648)		Apple leaves (SRM 1515)		Orchid leaves (SRM 1571)	
	Exp.	Cert.	Exp.	Cert.	Exp.	Cert.	Exp.	Cert.
Na	139 ± 5.9	152 ± 10	4028 ± 21	4250 ± 20	29 ± 1	24 ± 1	81 ± 0.7	82 ± 6
K%			1.0 ± 0.05	1.1 ± 0.01			1.4 ± 0.02	1.5 ± 0.07
Al					277 ± 2	286 ± 9		
S%	4.5 ± 0.1	4.3 ± 0.2						
Cl	26 ± 4	35*			564 ± 6	579 ± 23		
V	<0.2	0.03*			0.26 ± 0.03	0.3 ± 0.03		
Mn	6 ± 0.2	6.3 ± 0.5			52 ± 0.2	54 ± 3		
Mg					2583 ± 15	2710 ± 80		
Ca%					1.4 ± 0.02	1.5 ± 0.02		
Cu	10 ± 0.8	10.6 ± 0.7	545 ± 19	609 ± 27	7 ± 0.6	5.6 ± 0.2	12 ± 0.7	12 ± 1
Zn	187 ± 12	190 ± 5	4305 ± 53	4760 ± 140			22 ± 0.6	25 ± 2
Br	0.3 ± 0.01	0.4*	494 ± 3	500 ± 25	1.2 ± 0.1	1.8	9 ± 0.1	10 ± 1
Sr	21 ± 3	24 ± 1		207 ± 15			30 ± 4	37 ± 3
I	0.7 ± 0.1	0.8*						
Ba	17 ± 0.4	17 ± 1	667 ± 92	737 ± 40	44 ± 1	49 ± 2	42 ± 4	44 ± 4
Dy	<0.03	0.017						
U	0.1 ± 0.001	0.09*						
As	0.3 ± 0.001	0.28 ± 0.04	115 ± 1	115 ± 10			11 ± 0.1	10 ± 1
Cd	<4	0.11 ± 0.02	79 ± 9	75 ± 7				0.1 ± 0.02
Sb	0.082 ± 0.004	0.1 ± 0.01	46 ± 2	45 ± 2			3 ± 0.1	3 ± 0.3
La	0.055 ± 0.006	0.05 ± 0.01	36 ± 0.5	44 ± 2			1 ± 0.02	
Sm	0.012 ± 0.002	0.012*	4 ± 0.03	4 ± 0.3			0.1 ± 0.001	
Au	0.003 ± 0.001	0.0025*						
Sc	8 ± 0.1**	8 ± 1**	6 ± 0.2	7 ± 0.1			0.1 ± 0.002	0.1 ± 0.01
Cr	<0.260	0.37 ± 0.05	357 ± 9	403 ± 12			2.2 ± 0.1	2.6 ± 0.3
Fe	52 ± 6	54 ± 6*	3.7 ± 0.1%	3.9 ± 0.1%			252 ± 11	300 ± 28
Co	0.07 ± 0.006	0.07 ± 0.01	16 ± 0.4	18 ± 2			0.14 ± 0.01	0.16 ± 0.04
Se	0.60 ± 0.06	0.60 ± 0.03	25 ± 9	27 ± 1				0.08 ± 0.01
Ag	<0.07	0.029 ± 0.006	6 ± 0.3	6 ± 0.2				
Ce	0.15 ± 0.01	0.12 ± 0.03	56 ± 1	55 ± 4			1 ± 0.1	1 ± 0.1

*Not certified.

**ng/g.

**Table 2:** The concentration of elements in *μ*g/g (mean ± Std. error mean) found in hair children living in Dar es Salaam.

Elements	Mlimani (*n* = 16)	Kiwalani (*n* = 22)
Na	15.93 ± 2.71	31.17 ± 6.14
Mg	74 ± 13	113 ± 26
Al	103 ± 14	72 ± 14
S	46114 ± 577	46827 ± 582
Cl	1305 ± 142	1274 ± 192
K	44.78 ± 6.00	30.51 ± 4.93
Ca	447 ± 36	490 ± 48
V	0.18 ± 0.02	0.15 ± 0.03
Mn	4.88 ± 1.00	7.77 ± 1.24
Cu	10.64 ± 1.54	8.37 ± 0.53
Zn	104 ± 9	107 ± 4.81
Br	6.29 ± 0.87	6.34 ± 0.78
Sr	28.04 ± 2.25	7.42 ± 1.50
I	3.55 ± 1.03	6.74 ± 0.88
Ba	11.62 ± 2.31	6.67 ± 0.51
U	0.062 ± 0.003	0.05 ± 0.01
As	0.22 ± 0.06	0.17 ± 0.02
Sb	0.30 ± 0.06	0.40 ± 0.10
La	0.13 ± 0.03	0.29 ± 0.11
Sm	0.023 ± 0.004	0.013 ± 0.002
Au	0.13 ± 0.08	0.16 ± 0.04
Sc	0.014 ± 0.002	0.009 ± 0.002
Cr	0.40 ± 0.06	0.33 ± 0.05
Fe	59 ± 7	50 ± 9
Co	0.10 ± 0.02	0.19 ± 0.07
Se	0.49 ± 0.02	0.84 ± 0.36
Ag	0.15 ± 0.02	0.24 ± 0.03
Ce	0.31 ± 0.03	0.02 ± 0.17
Eu	0.025 ± 0.001	0.116 ± 0.002
